# Evaluation of Human Cerebrospinal Fluid Malate Dehydrogenase 1 as a Marker in Genetic Prion Disease Patients

**DOI:** 10.3390/biom9120800

**Published:** 2019-11-28

**Authors:** Inga Zerr, Anna Villar-Piqué, Vanda Edit Schmitz, Anna Poleggi, Maurizio Pocchiari, Raquel Sánchez-Valle, Miguel Calero, Olga Calero, Inês Baldeiras, Isabel Santana, Gabor G. Kovacs, Franc Llorens, Matthias Schmitz

**Affiliations:** 1 Department of Neurology, National Reference Center for CJD Surveillance University Medical Center Göttingen, 37075 Göttingen, Germany; vandaedit@gmail.com; 2 German Center for Neurodegenerative Diseases (DZNE)—Göttingen campus, 37075 Göttingen, Germany; 3 Bellvitge Biomedical Research Institute (IDIBELL), 08908 Hospitalet de Llobregat, Spain; 4 Network Center for Biomedical Research in Neurodegenerative Diseases, (CIBERNED), Instituto de Salud Carlos III, 28031 Madrid, Spain; mcalero@isciii.es (M.C.); rueda@externos.isciii.es (O.C.); 5 Department of Neurosciences, Istituto Superiore di Sanità, 00161 Rome, Italy; anna.poleggi@iss.it (A.P.); maurizio.pocchiari@iss.it (M.P.); 6 Alzheimer’s Disease and Other Cognitive Disorders Unit, Neurology Department, Hospital Clinic, IDIBAPS, 08036 Barcelona, Spain; 31799rsd@comb.cat; 7 Research Program on Digital Health, Chronicity and Healthcare Services (CROSADIS-UFIEC), Instituto de Salud Carlos III, 28220 Madrid, Spain; 8 Center for Neuroscience and Cell Biology, University of Coimbra, Coimbra, Portugal. Faculty of Medicine, University of Coimbra, 3004-517 Coimbra, Portugal; ines.baldeiras@sapo.pt (I.B.); isabeljsantana@gmail.com (I.S.); 9 Neurology Department, Centro Hospitalar e Universitário de Coimbra, 3004-561 Coimbra, Portugal; 10 Institute of Neurology, Medical University of Vienna, 1090 Vienna, Austria; gabor.kovacs@meduniwien.ac.at; 11 University of Toronto, Tanz Centre for Research in Neurodegenerative Disease, Toronto, ON M5S 3H2, Canada

**Keywords:** cerebrospinal fluid, diagnostic biomarker, genetic prion diseases, *PRNP* codon 129 MV genotypes, mitochondrial malate dehydrogenase 1

## Abstract

The exploration of accurate diagnostic markers for differential diagnosis of neurodegenerative diseases is an ongoing topic. A previous study on cerebrospinal fluid (CSF)-mitochondrial malate dehydrogenase 1 (MDH1) in sporadic Creutzfeldt–Jakob disease (sCJD) patients revealed a highly significant upregulation of MDH1. Here, we measured the CSF levels of MDH1 via enzyme-linked immunosorbent assay in a cohort of rare genetic prion disease cases, such as genetic CJD (gCJD) cases, exhibiting the E200K, V210I, P102L (Gerstmann–Sträussler–Scheinker syndrome (GSS)), or D178N (fatal familial insomnia (FFI)) mutations in the *PRNP*. Interestingly, we observed enhanced levels of CSF-MDH1 in all genetic prion disease patients compared to neurological controls (without neurodegeneration). While E200K and V210I carriers showed highest levels of MDH1 with diagnostic discrimination from controls of 0.87 and 0.85 area under the curve (AUC), FFI and GSS patients exhibited only moderately higher CSF-MDH1 levels than controls. An impact of the *PRNP* codon 129 methionine/valine (MV) genotype on the amount of MDH1 could be excluded. A correlation study of MDH1 levels with other neurodegenerative marker proteins revealed a significant positive correlation between CSF-MDH1 concentration with total tau (tau) but not with 14-3-3 in E200K, as well as in V210I patients. In conclusion, our study indicated the potential use of MDH1 as marker for gCJD patients which may complement the current panel of diagnostic biomarkers.

## 1. Introduction

Transmissible spongiform encephalopathies (TSE) belong to the group of neurodegenerative disorders characterized by the accumulation of misfolded prion protein resistant (PrP^Res^) in brain tissue. They can occur as sporadic, iatrogenic, or hereditary forms. Genetic prion diseases represent between 10 and 15% of all prion disease cases. More than 50 different mutations in the open reading frame of *PRNP* have already been described [1,2]. Depending on the mutation, the disease phenotype and neuropathological findings, different types of genetic prion diseases can be distinguished. The most common form is genetic Creutzfeldt–Jakob disease (gCJD) followed by fatal familial insomnia (FFI) and Gerstmann–Sträussler–Scheinker syndrome (GSS).

In addition to the *PRNP* mutation, the PrP gene encodes a polymorphism at codon 129 that influences the conformational structure of the cellular PrP (PrP^C^) [3], as well as disease course or the diagnostic accuracy of diagnostic tests [3,4,5,6,7].

Currently, the number and the accuracy of diagnostic test systems for prion diseases had been improved; however, autopsies are still required for a confirmation.

All biomarkers which are currently available, such as 14-3-3, total tau (tau), S-100 calcium-binding protein B, alpha-synuclein or neuron specific enolase, and the recently established in vitro protein misfolding amplification assays, real-time quaking-induced conversion (RT-QuIC), have a good accuracy for sporadic CJD (sCJD) diagnosis [8,9,10,11,12,13,14,15,16,17,18], but they have not yet been validated adequately for different groups of genetic prion diseases.

Previous studies of our group revealed a significant upregulation of mitochondrial malate dehydrogenase 1 (MDH1), an enzyme that reversibly catalyzes the oxidation of malate to oxaloacetate (part of many metabolic pathways, including the citric acid cycle) [19], in sCJD patients [20,21,22]. Best diagnostic accuracies were obtained by applying MDH1 in combination with other neurodegenerative markers [22].

These promising observations encouraged us to determine the levels of MDH1 in cerebrospinal fluid (CSF) of different types of genetic prion disease patients (gCJD (E200K, V210I), GSS, and FFI). We calculated the diagnostic sensitivity of MDH1, analyzed the influence of *PRNP* codon 129 MV polymorphism and estimated the association of MDH1 level with surrogate marker proteins, such as tau and 14-3-3.

## 2. Materials and Methods

### 2.1. Patient Population

We retrospectively analyzed 205 CSF samples obtained from patients recruited at the surveillance units from different participant centers (1) Clinical Dementia Center and the National Reference Center for CJD Surveillance at the University Medical Center, Goettingen, Germany, (2) Neurochemistry Laboratory, Neurology Department of Coimbra University Hospital, Coimbra, Portugal, (3) Alzheimer’s Disease and Other Cognitive Disorders Unit, Hospital Clinic, Barcelona, Spain, (4) National Centre of Microbiology-Carlos III Institute of Health, Madrid, Spain, (5) National Registry of CJD and related disorders at Istituto Superiore di Sanità, Rome, Italy, and (6) Institute of Neurology, Medical University of Vienna, Austria. The patients were classified according to established diagnostic criteria [23].

Our study was done in accordance with the Declaration of Helsinki and with informed written consent provided by all patients or by their next of kin in the case of cognitive impairment. Our study was approved by local ethic committees Goettingen (No. 24/8/12) and from ethical committees of other participating centers.

The cohort of patients in this study consisted of E200K-gCJD cases (*n* = 45), with *PRNP* mutation V210I-gCJD cases (*n* = 46), D178N-M-FFI (*n* = 36), P102L-GSS (*n* = 7), and neurological controls (NC) (*n* = 71) (Table 1). NC group included cases diagnosed with non-neurodegenerative, neurological, and psychiatric conditions according to acknowledged standard neurologic clinical and paraclinical findings based on the International Classification of Diseases and Related Health Problems, Tenth Edition definitions.

CSF samples were centrifuged for 10 min at 720× *g* (model no. 5415C centrifuge from Eppendorf, Hamburg, Germany) at room temperature (~22 °C), aliquoted in 1 mL portions and stored at −80 °C. Blood-stained CSF samples were excluded from the study. The analysis of the codon 129 MV genotype of *PRNP* was performed after isolation of genomic DNA from blood samples according to standard methods [24].

### 2.2. Determination of Human Malate Dehydrogenase 1

Levels of MDH1 were determined in CSF by a commercially available enzyme-linked immunosorbent assay (ELISA) kit (Cusabio, Hubei, China), following the manufacturer’s instructions. Briefly, levels of MDH1 were measured in arbitrary units (AU) per mL. The detection range of the assay spans from 9.38 to 600 AU/mL. To be in this range, CSF samples were measured in a dilution of 1:100. Colorimetric signal was analyzed at 450 nm with a 1420 Multilabel Counter Victor 2 (Wallac) (PerkinElmer, Waltham, MA, USA).

### 2.3. Determination of 14-3-3 Via Immunoblotting

We followed a slightly modified immunoblot protocol as described previously [25,26]. A polyclonal anti-14-3-3 β antibody (made against the human peptide sequence 14-3-3 β aa 1-100 (N terminal)), obtained from Abcam (Milton, UK) diluted 1:2000 was used as primary antibody. From seven isoforms, 14-3-3 β and γ are most suitable for CJD diagnostic [7].

As a secondary antibody we used a polyclonal horseradish peroxidase-conjugated antibody (Jackson Immuno Research, Leipzig, Saxony, Germany) (diluted 1:7500).

### 2.4. Determination of Tau in CSF Via ELISA

The CSF levels of protein tau were measured by a commercially available ELISA kit from INNOTEST hTAU-Ag (Fujirebio Europe, Gent, Belgium) with a detection range of total tau (phosphorylated and non-phosphorylated) between 50 and 2500 pg/mL (detection limit: 34 pg/mL). Briefly, before antibody incubations, each sample (75 µl) was diluted 1:1 in sample diluent. The colorimetric reaction was measured at 450 nm with a 1420 Multilabel Counter Victor 2 (Wallac) (PerkinElmer).

### 2.5. Statistical Analysis

Statistical data analysis was conducted by the use of the statistic software GraphPad Prism 6.01 (GraphPad Software, San Diego, CA, USA). For comparison between more than two groups, we used the Kruskal-Wallis test followed by Dunn’s post-hoc analysis. All correlation studies were computed by using the non-parametric Spearman’s correlation test (two-tailed) with a confidence interval of 95%. *p*-Values below 0.05 are considered as significant.

## 3. Results

### 3.1. Analysis of CSF-MDH1 Levels in gCJD Patients

In our study, we measured the MDH1 levels in CSF in different types of genetic prion diseases by using a commercial MDH-ELISA kit. Firstly, we excluded a potential sex-influence on MDH1 amount (Supplementary Figure S1).

**Figure 1:**
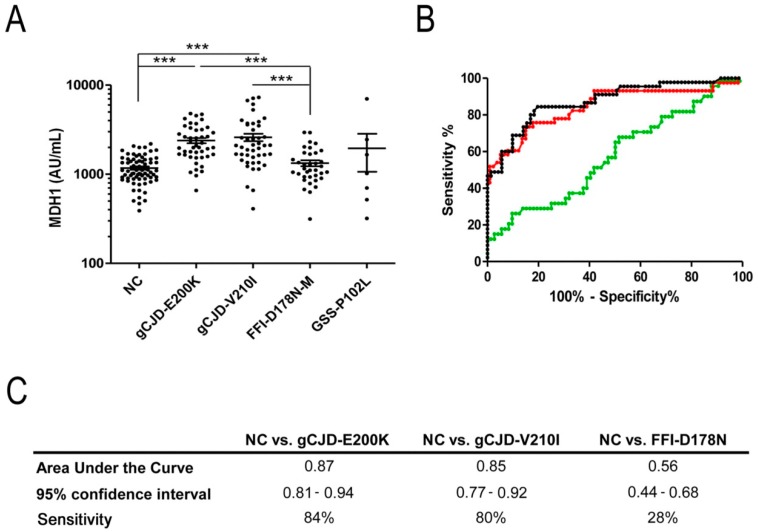
Detection of mitochondrial malate dehydrogenase 1 (MDH1) levels in cerebrospinal fluid (CSF) of genetic prion disease patients. (**A**) Patients with gCJD E200K (*n* = 45) and V210I (*n* = 46) contained a significantly increased level of MDH1 compared to fatal familial insomnia (FFI) cases (*n* = 36) and neurological controls (NC) (*n* = 71). GSS patients (*n* = 7) exhibited slightly higher MDH1 levels than FFI and controls, without statistical power due to a low number of samples. For comparison between groups we used Kruskal-Wallis test and Dunn’s post-hoc test. A *p*-value < 0.001 is considered as highly significant (***), <0.01 as very significant (**), <0.05 as significant (*), and ≥0.05 as not significant (ns). Values are indicated as arbitrary units (AU) per mL. Displayed are means ± SEM (standard error of the mean). (**B**) Receiver operating characteristic (ROC) curve analysis was performed to distinguish gCJD (E200K and V210I) patients and FFI from NC. Black, curve for gCJD-E200K; red, curve for gCJD-V210I; green, curve for FFI (**C**) area under the curve (AUC) values, corresponding to the area under ROC curves, and 95% confidence intervals are reported. Diagnostic sensitivity for gCJD patients was 0.85–0.87 AUC.

The analysis of MDH1 levels revealed a significant increase in genetic prion disease patients in comparison to a control group (*p* < 0.001 ***) (Table 1, Figure 1A). The comparative analysis of different genetic prion disease groups indicated higher MDH1 levels in gCJD (E200K and V210I) than in FFI cases (*p* < 0.001) (Figure 1A).

When we performed a receiver operating characteristic curve analysis for each group, the diagnostic accuracy to discriminate gCJD (E200K and V210I) from controls was 0.87 area under de curve (AUC), 95% Cl = 0.81–0.94, sensitivity 84% for E200K and 0.85 AUC, 95% CI = 0.77–0.92, sensitivity 80% for V210I (Figure 1B,C). In contrast, we found CSF-MDH1 levels inadequate to differentiate between FFI and control samples (AUC 0.56, 95% Cl = 0.44–0.68, sensitivity 28%) (Figure 1B,C).

**Figure 2:**
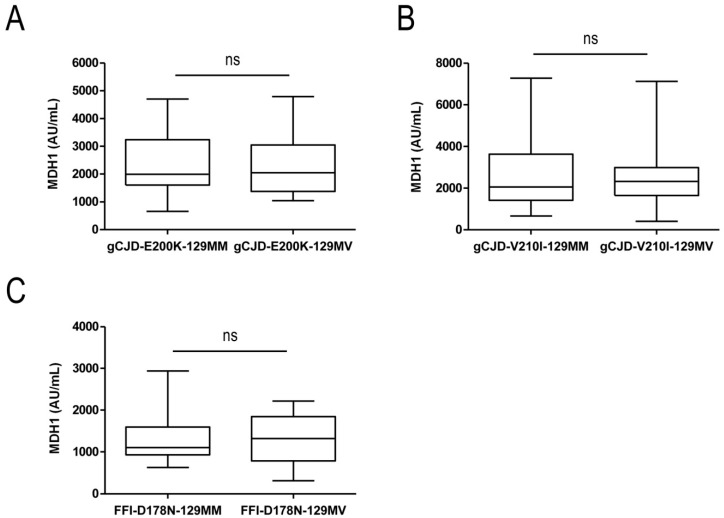
Comparative cerebrospinal fluid (CSF) mitochondrial malate dehydrogenase 1 (MDH1) analysis of different *PRNP* codon 129 genotypes. Genetic CJD E200K (**A**), V210I (**B**), and FFI (**C**) patients were stratified according to their *PRNP* codon 129 MV genotypes. Comparing CSF-MDH1 of *PRNP* codon 129 MM and MV carriers revealed no significant differences between the groups. Displayed are means ± SEM (standard error of the mean). ns, not significant.

To study the influence of the *PRNP* codon 129 MV polymorphism on MDH1-levels in CSF, we stratified our genetic prion disease cohort according to the *PRNP* codon 129 genotype in gCJD (E200K MM and MV, V210I MM and MV, as well as FFI-D178N MM and MV). In none of the groups we observed changes in the CSF-MDH1 levels depending on the codon 129 MV genotype, suggesting that this polymorphism has no impact on CSF-MDH1 concentration (Figure 2A–C).

### 3.2. Correlation between MDH1 and Other Neurodegenerative Markers

To explore a putative association between CSF-MDH1 levels and neurodegenerative biomarker proteins for prion diseases (tau and 14-3-3), we performed a correlation study.

**Figure 3:**
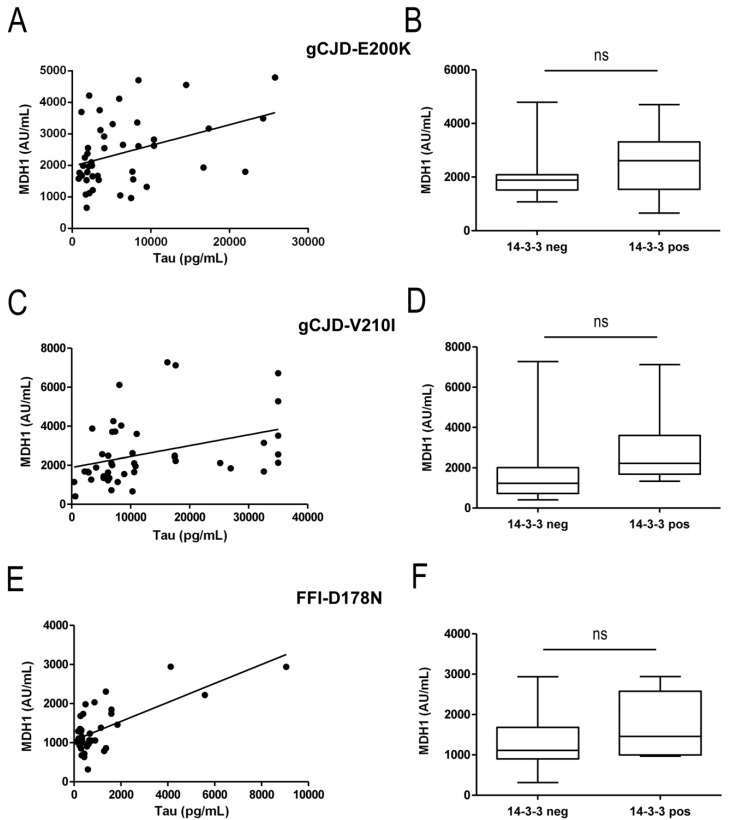
Correlation study of cerebrospinal fluid (CSF) mitochondrial malate dehydrogenase 1 (MDH1) levels with tau and 14-3-3 amounts. CSF-levels of MDH1, tau, and 14-3-3 from the same patients were correlated to detect potential association among neurodegenerative markers. (**A**–**F**). In gCJD (E200K and V210I) patients, a positive correlation was obtained between MDH1 and tau levels (E200K: *r* = 0.39; *p* < 0.01 **; *n* = 45), (V210I: *r* = 0.51; *p* < 0.01 **; *n* = 46). No correlation was detected between MDH1 and tau in FFI cases. MDH1 and 14-3-3 did not correlate at all.

Here, we determined the analyzed markers within the same samples. Interestingly, levels of CSF-tau correlated positively with MDH1 levels in E200K (*r* = 0.39; *p* < 0.01 **) and V210I (V210I: *r* = 0.51; *p* < 0.01 **) cases but not in FFI cases (Figure 3A,C,E).

The amount of 14-3-3 in CSF was detected by Western blot and considered either as 14-3-3 positive (14-3-3 band is visible) or as negative (14-3-3 band is not visible). The comparison of MDH1 levels from 14-3-3 positive and negative patients revealed for E200K, V210I, and FFI cases no significant association between MDH1 and 14-3-3 (Figure 3B,D,F).

All correlation studies were computed by using the non-parametric *Spearman’s* correlation test (two-tailed) with a confidence interval of 95%. A *p*-value < 0.001 is considered as highly significant (***), <0.01 as very significant (**), <0.05 as significant (*), and ≥0.05 as not significant (ns).

## 4. Discussion

Some *PRNP* point mutations, such as V210I or E200K, are associated with variable disease onsets (usually between 30 and 70 years) and penetrance [27,28]. The diagnostic accuracy of surrogate markers or tests in CSF, such as 14-3-3, tau, alpha-synuclein, or RT-QuIC as well as instrumental diagnostic techniques (EEG, FLAIR, and DWI MRI techniques) depends on the type of *PRNP* mutation [27,28,29]. In certain types of hereditary prion diseases, such as FFI or GSS, the diagnostic accuracy of these tests was less investigated than in sCJD patients. Due to rareness of patients, the study cohorts of genetic patients were usually limited, and several diagnostic tests were less accurate in genetic prion diseases (e.g., FFI) in comparison to sCJD [30,31], highlighting the relevance of biomarker research in different types of genetic prion diseases.

A previously conducted two-dimensional proteomic study on CJD brain homogenates and CSF, as well as a recent retrospective study, indicated a specific regulation of MDH1 in sCJD patients compared to controls [20,21,22].

These interesting observations encouraged us to investigate a potential regulation of MDH1 levels in CSF of genetic prion disease patients in comparison to a neurodegenerative control cohort. Moreover, we were also interested in comparing MDH1 levels with different genetic prion disease groups, such a gCJD E200K, V210I, FFI, and GSS.

Our data indicate an enhancement of MDH1 amount as a consequence of rapid neurodegeneration in CSF of genetic prion disease patients compared to controls. Within the genetic prion disease groups, gCJD E200K and V210I carriers showed the highest MDH1 levels in CSF with AUC value of 0.87 and 0.85 followed by GSS (without statistical power) and FFI cases (0.56 AUC, showing a poor discrimination accuracy), which revealed a dependency of MDH1 levels on the type of genetic prion disease. The diagnostic accuracy of MDH1 in gCJD is comparable to sCJD (specificity of 85% and a sensitivity of 83%) [22]. In the context of other surrogate biomarkers, such as 14-3-3, the sensitivity to diagnose E200K was indicated between 77 and 89% [32,33,34]. In addition, our recent study on alpha-synuclein using an electro-chemiluminescent assay based on the *Meso Scale Discovery* (MSD) technology, revealed a diagnostic accuracy for gCJD E200K of 0.96 AUC and for FFI of 0.75 AUC [35] which is higher than MDH1 detection.

A limitation of this analysis is the small number of GSS cases that did not allow proper statistics. Therefore, it was not possible to calculate the diagnostic accuracy for this group.

When we analyzed the impact of the *PRNP* codon 129 MV polymorphism (MM and MV) on MDH1 levels in genetic prion disease patients, we did not find any difference on MDH1 concentrations for any genotype. Our results are in agreement with a previous study [22], in which it was reported that the MDH1 levels do not depend on sCJD codon 129 MV genotype. In this context, other diagnostic biomarkers or assays, such as 14-3-3, tau or RT-QuIC also failed to distinguish accurately between different sCJD genotypes [30,36,37].

Correlation studies of MDH1 levels with other neurodegenerative marker proteins, such as tau or 14-3-3 revealed a significant correlation only between MDH1 and tau in gCJD (E200K and V210I) but not for FFI, which most probably depend on the low upregulation of MDH1 in this group. As opposed to previous observations on sCJD patients, in our present study on genetic prion diseases, we observed no correlation between MDH1 and 14-3-3. This disagreement may be explained by the detection method for 14-3-3 [11]. Unfortunately, only Western blot data on 14-3-3 detection, but no ELISA data were available.

For a further validation of CSF-MDH1 as a protein marker for genetic prion disease diagnosis.

MDH1 levels should be determined in a larger patient cohort, which might be challenging due to the rareness of genetic prion disease cases worldwide.

## 5. Conclusions

In the present study we determined the levels of CSF-MDH1 in a cohort of genetic prion disease patients. Our data indicated an upregulation of MDH1 levels in gCJD (E200K and V210I) patients (with a sensitivity of 80–84%) recommending MDH1 either alone or in combination with other tests as a potential biomarker for gCJD (E200K, V210I) diagnosis.

## Figures and Tables

**Table 1:** Characteristics of the patient population analyzed in the present study. Biomarker MDH1 and tau data are indicated as mean values ± standard deviation, while we considered 14-3-3 β, detected by Western blotting either as positive or negative.

			Age (years)	MDH1 (AU/mL)		Tau (pg/mL)	14-3-3	*p*-Value	
	*n*	Sex (f/m)	Mean +/− SD	Mean +/− SD	95% CI	Mean +/− SD	Pos/Neg	MDH1 vs. Sex	MDH1 vs. Age
NC	71	35/36	61± 9	1172 ± 413	1075–1270	304 ± 212 *	NA	0.37	0.12
gCJD—E200K	45	28/17	59 ± 11	2385 ± 1066	2065–2705	6263 ± 6310	40/5	0.84	0.19
gCJD—V210I	46	24/22	63 ± 9	2595 ± 1669	2100–3091	12710 ± 10815	37/9	0.45	0.31
FFI—D178N-M	36	12/24	51 ± 10	1330 ± 606	1125–1535	1122 ± 1741	5/31	0.79	0.24
GSS—P102L	7	3/4	52 ± 10	1954 ± 2348	0–4125	1337 ± 1264	0/7	0.85	0.9

MDH1, mitochondrial malate dehydrogenase 1; *n*: number of cases; f/m: female/male; SD: standard deviation; 95% CI: 95% confidence interval; Pos/Neg: positive/negative, inconclusive 14-3-3 tests were considered as negative, * 10 values were not available; NC, neurological controls; gCJD, Creutzfeldt–Jakob disease, FFI, fatal familial insomnia; GSS, Gerstmann–Sträussler–Scheinker syndrome.

## Supplementary Materials

The following are available online at https://www.mdpi.com/2218-273X/9/12/800/s1, Figure S1: CSF- MDH1 levels in females and males.
