# Supplementary material for: Evaluation of Human Cerebrospinal Fluid Malate Dehydrogenase 1 as a Marker in Genetic Prion Disease Patients
Source: Biomolecules. 2019 Nov 28;9(12):800. doi: 10.3390/biom9120800 (PMC6995564; doi:10.3390/biom9120800)
Supplement: Supplementary file 1 [file biomolecules-09-00800-s001.pdf]

biomolecules

Supplementary Materials

Evaluation of Human Cerebrospinal Fluid Malate Dehydrogenase 1 as a Marker in Genetic Prion Disease Patients

Inga Zerr [1,2,*,†], Anna Villar-Piqué [3,4,*,†], Vanda Edit Schmitz [1], Anna Poleggi [5], Maurizio Pocchiari [5], Raquel Sánchez-Valle [6], Miguel Calero [4,7], Olga Calero [4], Inês Baldeiras [8], Isabel Santana [8], Gabor G. Kovacs [10,11], Franc Llorens [1,3,4,*,†] and Matthias Schmitz [1,2,*,†]

[1] Department of Neurology, National Reference Center for CJD Surveillance University Medical Center Göttingen and German Center for Neurodegenerative Diseases (DZNE)—Göttingen campus, 37075 Göttingen, Germany
[3] Bellvitge Biomedical Research Institute (IDIBELL), 08908 Hospitalet de Llobregat, Spain.
[4] Network Center for Biomedical Research in Neurodegenerative Diseases, (CIBERNED), Instituto de Salud Carlos III, Madrid 28031, Spain
[5] Department of Neurosciences, Istituto Superiore di Sanità, 00161 Rome, Italy
[6] Alzheimer's Disease and Other Cognitive Disorders Unit. Neurology Department. Hospital Clinic. IDIBAPS. 08036 Barcelona, Spain
[7] Research Program on Digital Health, Chronicity and Healthcare Services (CROSADIS-UFIEC), Instituto de Salud Carlos III, Madrid 28220, Spain.
[8] Center for Neuroscience and Cell Biology, University of Coimbra; Faculty of Medicine, University of Coimbra, 3004-517 Coimbra, Portugal
[9] Neurology Department, CHUC—Centro Hospitalar e Universitário de Coimbra, CNC—Center for Neuroscience and Cell Biology, Faculty of Medicine, University of Coimbra, 3004-561 Coimbra, Portugal.
[10] Institute of Neurology, Medical University of Vienna, 1090 Vienna, Austria
[11] University of Toronto, Tanz Centre for Research in Neurodegenerative Disease, M5S 3H2 Toronto, Ontario, Canada
* Correspondence: ingazerr@med.uni-goettingen.de (I.Z.); avillar@gwdg.com (A.V.-P.); franc.llorens@gmail.com (F.L.); matthias.schmitz@med.uni-goettingen.de (M.S.)
† These authors equally contributed to this work

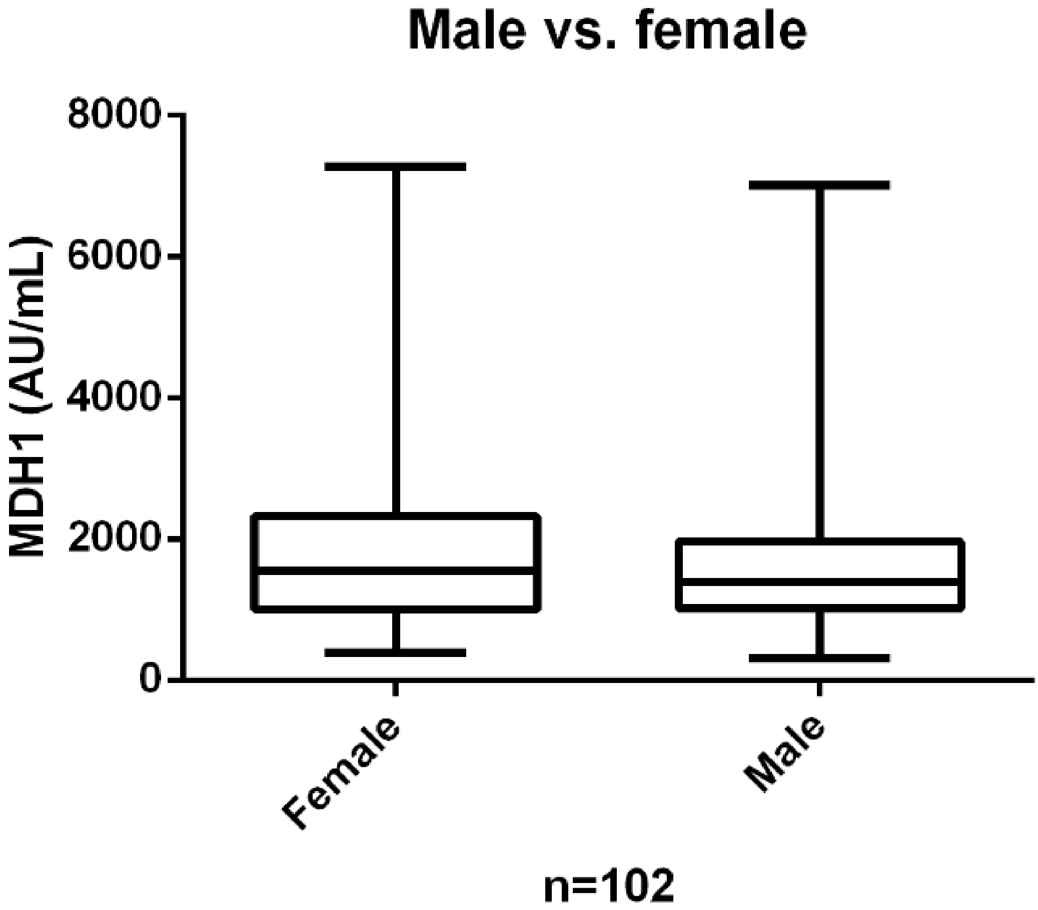

Figure S1. CSF MDH1 levels in females and males.
